# Identification of Phage Viral Proteins With Hybrid Sequence Features

**DOI:** 10.3389/fmicb.2019.00507

**Published:** 2019-03-26

**Authors:** Xiaoqing Ru, Lihong Li, Chunyu Wang

**Affiliations:** ^1^School of Information and Electrical Engineering, Hebei University of Engineering, Handan, China; ^2^School of Computer Science and Technology, Harbin Institute of Technology, Harbin, China

**Keywords:** phage virion proteins, machine learning, feature extraction, feature selection, hybrid sequence features

## Abstract

The uniqueness of bacteriophages plays an important role in bioinformatics research. In real applications, the function of the bacteriophage virion proteins is the main area of interest. Therefore, it is very important to classify bacteriophage virion proteins and non-phage virion proteins accurately. Extracting comprehensive and effective sequence features from proteins plays a vital role in protein classification. In order to more fully represent protein information, this paper is more comprehensive and effective by combining the features extracted by the feature information representation algorithm based on sequence information (CCPA) and the feature representation algorithm based on sequence and structure information. After extracting features, the Max-Relevance-Max-Distance (MRMD) algorithm is used to select the optimal feature set with the strongest correlation between class labels and low redundancy between features. Given the randomness of the samples selected by the random forest classification algorithm and the randomness features for producing each node variable, a random forest method is employed to perform 10-fold cross-validation on the bacteriophage protein classification. The accuracy of this model is as high as 93.5% in the classification of phage proteins in this study. This study also found that, among the eight physicochemical properties considered, the charge property has the greatest impact on the classification of bacteriophage proteins These results indicate that the model discussed in this paper is an important tool in bacteriophage protein research.

## Introduction

In the biological world, bacteriophages are ubiquitous, with different genomes and lifestyles. According to their morphology, they can be classified as either tail, tailless, or filamentous bacteriophages. According to morphology and nucleic acid, phages are classified as infect bacteria and infect archaea. The bacteriophage must be attached to a host cell for growth and reproduction (Seguritan et al., 2012), and directly affects the host population by lysing host cells. In addition, each bacteriophage is specific and greatly reduces the damage to host cells (Haq et al., 2012). Identification and classification of various bacteria can be performed based on the universality, diversity, dependence, and specificity of bacteriophages (Marks and Sharp, 2015). The structure of bacteriophages is simple, consisting of only a protein shell and genetic material (DNA or RNA) (Haq et al., 2012), making them important substances for simplifying experimental research in bioinformatics. As a bacteriophage can insert genes into host cells (Ding et al., 2014), it is an important tool for studying genetics (Cheng et al., 2018; Hu et al., 2018). Hershey (Hershey and Chase, 1952) performed biological experiments using the T2 bacteriophage and bacteria in 1952, and finally confirmed that DNA is the genetic material of bacteriophages and other organisms. The significance of this research in the development of biological science earned Hershey and coworkers the Nobel Prize in Physiology. Bacteriophage provide experimental systems and tools for the molecular biological science revolution. The bacteriophage rapid development has led to dection of basic principles of ecology and evolution. Besides, it is relatively easy to synthesize and has modular characteristic, which cater to the needs of synthetic biologists and carry out engineering research and implementation of biological function.

Bacteriophage proteins are classified into virion and non-viron proteins (Zhang et al., 2015), with most practical interest focusing on the function of bacteriophage virion proteins (Feng et al., 2013b). Therefore, bacteriophage proteins must be accurately classified and identified so that researchers can further study the structure and function of a particular bacteriophage. After the human genome project was officially launched in 1990, the number of bacteriophage protein sequences with unknown functions increased dramatically (Seguritan et al., 2012; Chen et al., 2018a). Faced with a large volume of data, traditional biological experimental methods could no longer keep up with the post-gene era (Chen W. et al., 2016; Cheng et al., 2019; Mrozek et al., 2016; Hu et al., 2018). For this reason, researchers introduced different machine learning algorithms into bacteriophage classification and prediction research. For example, Li et al. (2007) developed a support vector machine system called SynFPS that uses the gene–gene distance determined by k-means clustering to identify closely related genomes and perform gene function prediction. Using the protein appearance frequency of amino acids and information of isoelectric points, Seguritan et al. (2012) developed an artificial neural network method to classify viral structures. Feng et al. (2013b) used the main amino acid and dipeptide components as an encoding scheme, and modified a naive Bayes classifier to identify bacteriophage proteins. Ding et al. (2014) used g-gap dipeptide composition to represent protein sequence information, incremental feature selection to analyze the variance and identify the optimal feature set, and a support vector machine for classification. Zhang et al. (2015) obtained sequence feature vectors with various techniques, and then used the incremental feature selection algorithm to select the optimal feature subsets. Finally, the prediction results of individual classifiers trained in different feature spaces were integrated to produce the final classification effect. Machine learning algorithm (Robert, 2012; Stephenson et al., 2018) automatically analyze and obtain rules from data and use them to predict unknown data (Chen and Yan, 2013; Yu et al., 2015, 2016a; Chen and Huang, 2017; Chen et al., 2018h; Wang et al., 2018). This saves time and money, but the results from such algorithms are not as convincing as those from biological experiments. Therefore, it is especially important to choose an appropriate machine learning algorithm to ensure the most accurate classification results (Liu, 2017; Yao et al., 2017; Yu et al., 2017a). In a protein classification experiment, the classification effect depends largely on the feature set extracted (Zou et al., 2013; Bin et al., 2015; Mrozek et al., 2015; Jia et al., 2016; Yu et al., 2016b, 2018; Zhang et al., 2016; Huang et al., 2017; Qu et al., 2017; Jiang et al., 2018; Qiao et al., 2018; Xiong et al., 2018; Xu et al., 2018b). To date, feature extraction methods are divided into sequence-based and structure-based approaches (Huang et al., 2017; Qu et al., 2017) The feature set extraction part of this study is obtained by combining the features extracted by the two feature extraction methods.

**Figure 1:**
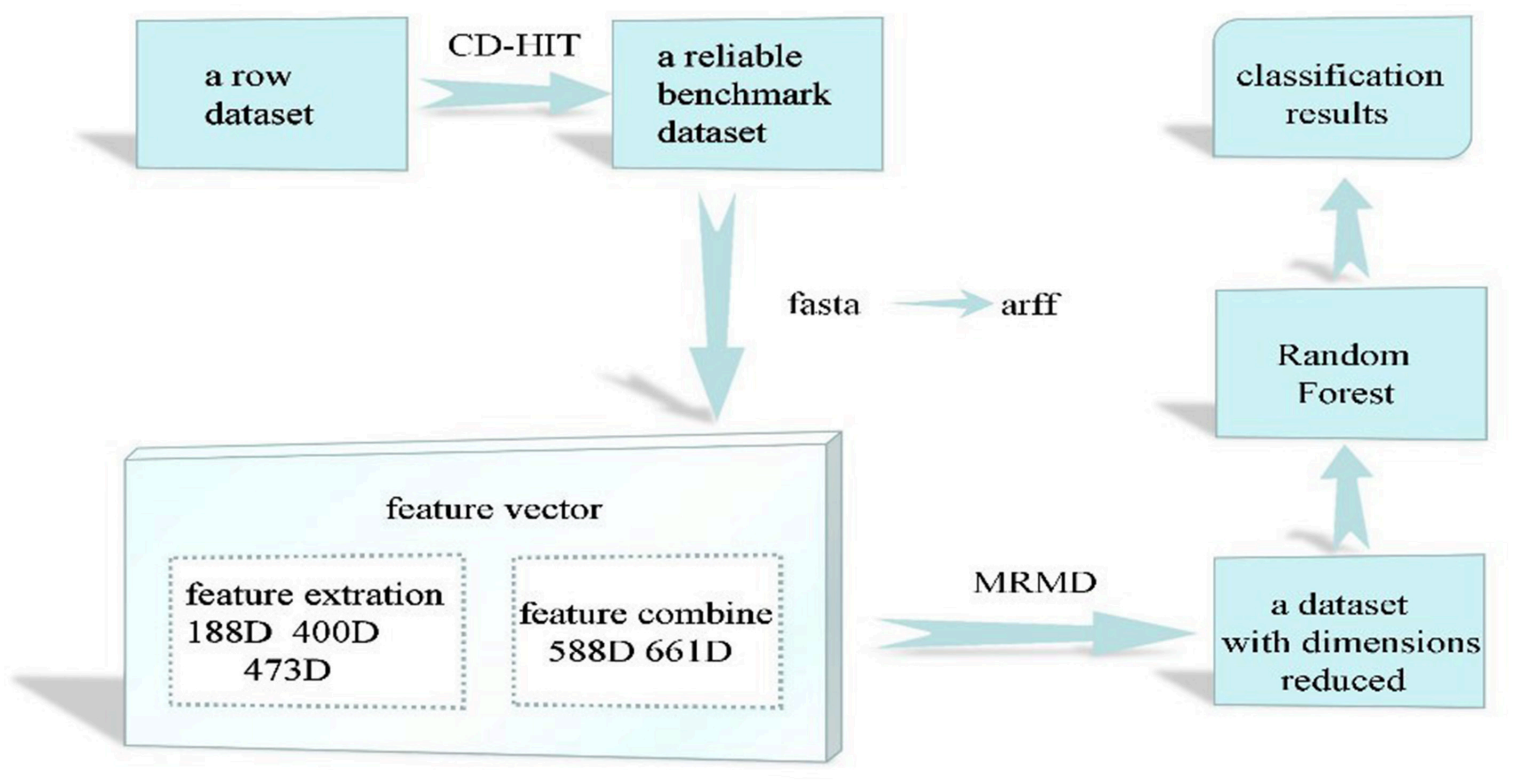
Outline flowchart of this study.

In this study, we examined the final classification effect of the selected methods and the stability of the dataset when the feature dimension was reduced. First, to remove the imbalance in the reference dataset, CD-Hit was used to remove redundant data, resulting in a balanced dataset that contains comprehensive information and less redundancy. Pearson's correlation coefficient and three distance functions (Euclidean and cosine distances and the Tanimoto coefficient) (Zou et al., 2016) were then used to calculate the correlation between features and class labels and the redundancy between features. Finally, the optimal feature subset with the strongest correlation between features and class labels and low redundancy between features was selected. According to some recent studies(Wu et al., 2009; Yi et al., 2011; Chen and Lin, 2012; Yang et al., 2015; Yu et al., 2017b; Zhang and Liu, 2017; Xu et al., 2018a; Liu et al., 2019), the best algorithms for protein classification are support vector machines and random forest algorithms. However, support vector machines are more suitable for small sample sets in which the number of dimensions is greater than the number of samples. Thus, the random forest algorithm was used in this study. The random forest algorithm (Breiman, 2001; Yao et al., 2017) combines multiple weak classifiers to produce a final result that has higher accuracy and better generalization performance. It can achieve good results, mainly because of the random nature of the “forest,” which makes the algorithm resistant to overfitting and more precise. Finally, in terms of bacteriophage protein classification, the data set extracted by combining the features and the feature selection of the feature set have a positive impact on the protein classification effect. Our results also show that, among the eight physicochemical properties of amino acids, the charge property has the greatest influence on the classification of bacteriophage proteins. To evaluate the performance of the models used in this study, the results were compared with those given by the methods introduced in (Feng et al., 2013b; Ding et al., 2014; Zhang et al., 2015). Figure 1 shows the workflow of this study.

## Methods

### Dataset Processing

Source: UniProt (Rolf, 2004; Consortium, 2012) is a widely used protein sequence database that offers low protein sequence redundancy and complete protein function interpretation (Cao and Cheng, 2016a; Jiang et al., 2016). As this website is free and open, researchers can download the desired protein sequence for free. The original positive samples used in this study (a total of 15,765 data), e.g., the number of bacteriophage virion proteins, were downloaded from this database. After obtaining the bacteriophage virion protein (positive) sample set, the PFAM family of positive samples was excluded from all PFAM families, such that the remaining samples were families of non-phage virion proteins. Finally, the longest protein sequence of the remaining families was extracted to form a negative sample set. The positive and counterexample datasets obtained as described above may all contain homologous sequences. Using such sample sets would result in the classification accuracy being overestimated, which is not conducive to the establishment of prediction models. Therefore, we used the CD-Hit tool to remove redundant positive and negative samples from the datasets.

Data integration: The CD-Hit (Li et al., 2001; Li and Godzik, 2006; Huang et al., 2010; Fu et al., 2012; Chen et al., 2017) redundancy tool effectively clusters similar sequences. The basic principle is to sort protein sequences in the dataset in descending order. The longest sequence is taken as the first class, and then this is compared with the second-longest protein sequence in terms of their similarity. If the similarity between the two is greater than some threshold, they are deemed to belong to the same class. Otherwise, the second-longest sequence forms a new class. Because the bacteriophage virion protein sequences were downloaded from UniProt, which ensures relatively low redundancy, the interrupt threshold was set to 0.8. The non-phage virion proteins had a higher degree of redundancy, so their interrupt threshold was set to 0.4. Thus, 6,251 bacteriophage virion protein sequences and 9,514 non-phage virion protein sequences were obtained. The union of the resulting positive and negative sample datasets gives the total dataset, and the intersection of the two is empty.

### Feature Extraction

#### Representation Algorithms for Amino Acid Composition and Eight Physicochemical Properties

In this study, a feature set containing 188 dimensions was extracted based on amino acid composition and eight physicochemical properties. The amino acid composition is one of the most basic features of proteins (Zhang et al., 2015; Cao and Cheng, 2016b). Eight physicochemical properties of amino acids also play a role in the functional properties of bacteriophage proteins. In 1988, Coia et al. (1988) found that amino acids having lighter side chain groups are more likely to constitute bacteriophage virion sequences. In 1994, Marvin et al. (1994) proposed that hydrophilicity, hydrophobicity, and charge have a greater impact on the function of bacteriophage virion proteins. In 2008, Shen and Chou (2008) identified the vital role that the hydrophilicity and hydrophobicity of amino acids play in the folding of proteins. In 2014, Ting et al. (2014) used logistic regression to integrate several biological features, including physicochemical properties for predicting lysine acetylation, thus demonstrating the effect of physicochemical properties on protein structure and function. Therefore, the amino acid composition and its eight physicochemical properties are used to extract features that reflect the characteristics of bacteriophage proteins.

The 20 most common amino acids are as follows:

(1) $$\begin{array}{l} CAA=\left\{A,C,D,E,F,G,H,I,K,L,M,N,P,Q,R,S,T,V,W,Y\right\} \end{array}$$

The occurrence frequency of each amino acid in a protein sequence can be expressed as:

(2) $$\begin{array}{l} f_{1i}=\left\{\frac{n_{i}}{L}|1\leq i\leq 20\right\} \end{array}$$

Where *n*_*i*_ is the frequency with which amino acid *i* occurs in the protein sequence and *L* is the length of the protein sequence.

**Figure 2:**
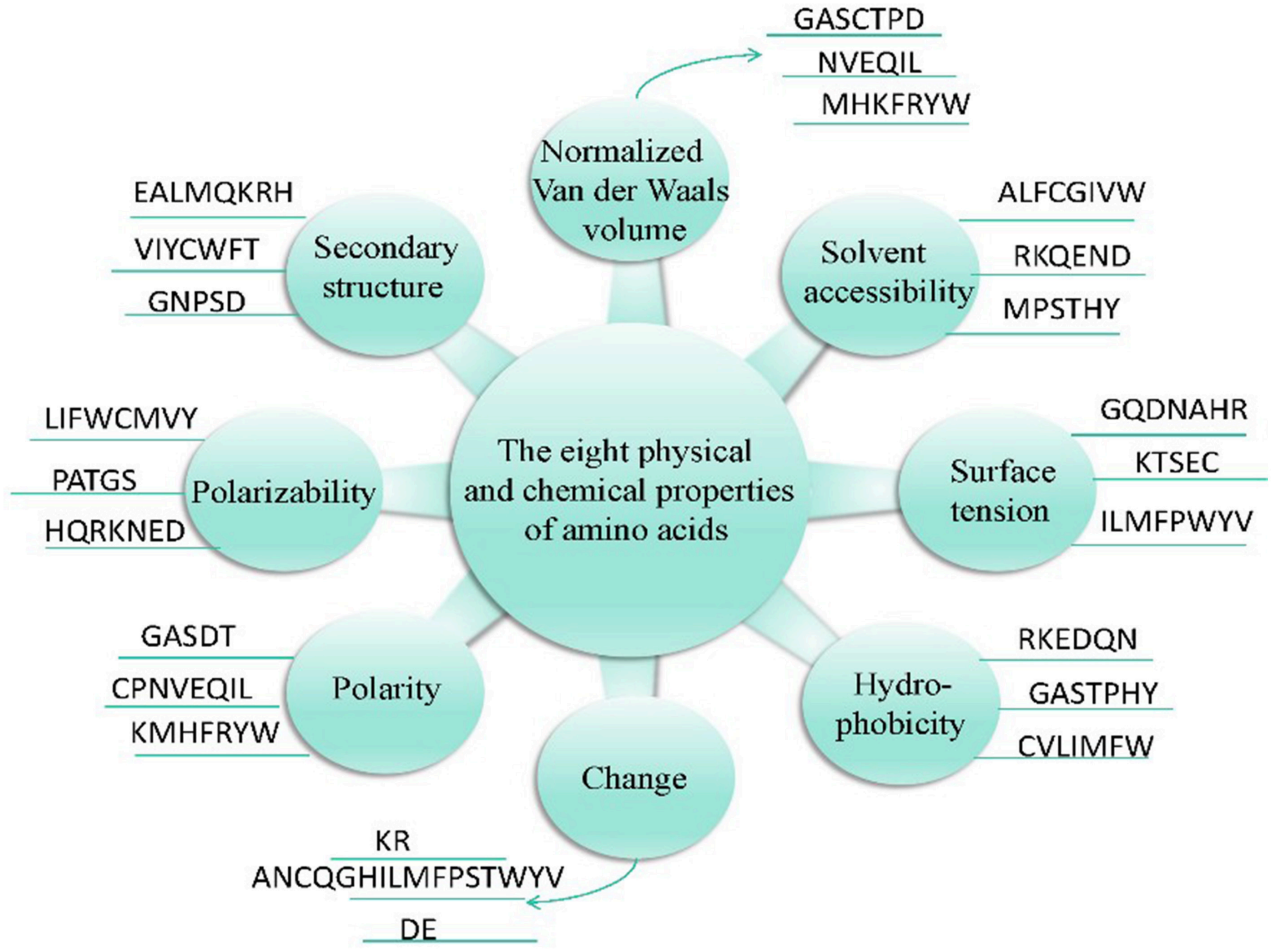
Eight physicochemical properties of amino acids.

In addition, these 20 amino acids can be classified into three types according to their physicochemical properties (Chou and Com, 2010), as shown in Figure 2.

The composition, transformation, and distribution of amino acids were determined by Dubchak et al. (1995) based on a global description of protein sequences. The feature extraction methods for the eight physicochemical properties of a protein sequence are as follows. Taking the electrode polarity as an example (expressed by *p*), the 20 amino acids are divided into high-, medium-, and low-charged polarity groups, which are expressed by *p*_*h*_, *p*_*p*_, *p*_*l*_, respectively. The composition, transformation, and distribution of the amino acids at this time can be represented by equations (3)–(7).

Composition features (Dubchak et al., 1995) (frequency of each charged electrode group in a sequence):

(3) $$\begin{array}{l} \left(f_{21},f_{22},f_{23}\right)=\left[\frac{n_{1}p_{h}}{L},\frac{n_{2}p_{p}}{L},\frac{n_{3}p_{l}}{L}\right] \end{array}$$

where *f*_21_, *f*_22_, *f*_23_ denote the content of the high-, medium-, and low-charged polarity groups in a sequence, respectively,*L* is the length of the protein sequence,*n*_1_, *n*_2_, *n*_3_ are the frequencies with which the three electrode groups appear in the sequence.

Conversion feature (Dubchak et al., 1995) (frequency of occurrence of bigeminal sequences):

(4) $$\begin{array}{l} \left(f_{31},f_{32},f_{33}\right)=\left[\frac{m_{1}p_{hl}}{L-1},\frac{m_{2}p_{hp}}{L-1},\frac{m_{3}p_{pl}}{L-1}\right] \end{array}$$

Where *f*_31_, *f*_32_, *f*_33_ denote the content of the three bigeminal groups *p*_*hl*_, *p*_*hp*_, *p*_*pl*_, and *m*_1_, *m*_2_, *m*_3_ are the frequencies of these three bigeminal groups appearing in sequence. There are three possible sequences of the charged polarity: *p*_*hl*_, *p*_*hp*_, *p*_*pl*_ In addition, in a protein sequence of length *L*, assuming that any two adjacent amino acids constitute a pair, the protein sequence contains *L* − 1 paired sequences (Zou et al., 2013).

Distribution features (Dubchak et al., 1995) (amino acid distribution of the high-, medium-, and low-charged polarity groups):

(5) $$\begin{array}{l} {\left(f_{411},f_{412},f_{413},f_{414},f_{415}\right)}^{T}={\left[a_{1\%},a_{25\%},a_{50\%},a_{75\%},a_{100\%}\right]}^{T} \end{array}$$

(6) $$\begin{array}{l} {\left(f_{421},f_{422},f_{423},f_{424},f_{425}\right)}^{T}={\left[b_{1\%},b_{25\%},b_{50\%},b_{75\%},b_{100\%}\right]}^{T} \end{array}$$

(7) $$\begin{array}{l} {\left(f_{431},f_{432},f_{433},f_{434},f_{435}\right)}^{T}={\left[c_{1\%},c_{25\%},c_{50\%},c_{75\%},c_{100\%}\right]}^{T} \end{array}$$

Where *a*_1%_, *a*_25%_, *a*_50%_*a*_75%_*a*_100%_ represent the positions of the first, 25, 50, 75, and 100% high-charged polarity groups in a sequence, *b*_1%_, *b*_25%_, *b*_50%_, *b*_75%_, *b*_100%_ represent the positions of the first, 25, 50, 75, and 100% medium-charged polarity groups in a sequence and *c*_1%_, *c*_25%_, *c*_50%_, *c*_75%_, *c*_100%_ represent the positions of the first, 25, 50, 75, and 100% low-charged polarity groups in a sequence.

In summary, (3 + 3 + 3 × 5) = 21-dimensional features can be extracted from each physicochemical property, and so 8 × 21 = 168-dimensional features can be extracted from the eight physicochemical properties. The 188-dimensional features (20-dimensional + 168-dimensional) are used to express the characteristics of bacteriophage proteins, and are extracted based on the content ratio of each of the 20 amino acids in the sequence and the eight physicochemical properties.

#### Adaptive k-skip-*n*-Gram Algorithm

A feature set containing 400 dimensions is extracted based on the adaptive k-skip-n-gram method (Feng et al., 2013c; Cao et al., 2017; Wei et al., 2017a; Tang et al., 2018). In this study, the value of *n* was set to 2 (20^2^ = 400).

The *K* value represents the separation distance between two amino acids. For example, in the protein sequence *S* = *A*_1_*A*_2_*A*_3_⋯*A*_*L*_ (where *L* is the length of the sequence),

(8) $$\begin{array}{l} K=i-j-1 \end{array}$$

And *A*_*i*_,*A*_*j*_ are the *i*th and *j*th amino acids of *S*.

In a bacteriophage protein dataset, the sequences have very different lengths. If the parameter *K* is fixed to a specific value, the sequence information cannot be properly represented, which will affect the final classification effect. Therefore, the value of *k* was set to be adaptive so that *K* could vary with the length of the sequence.

**Figure 3:**
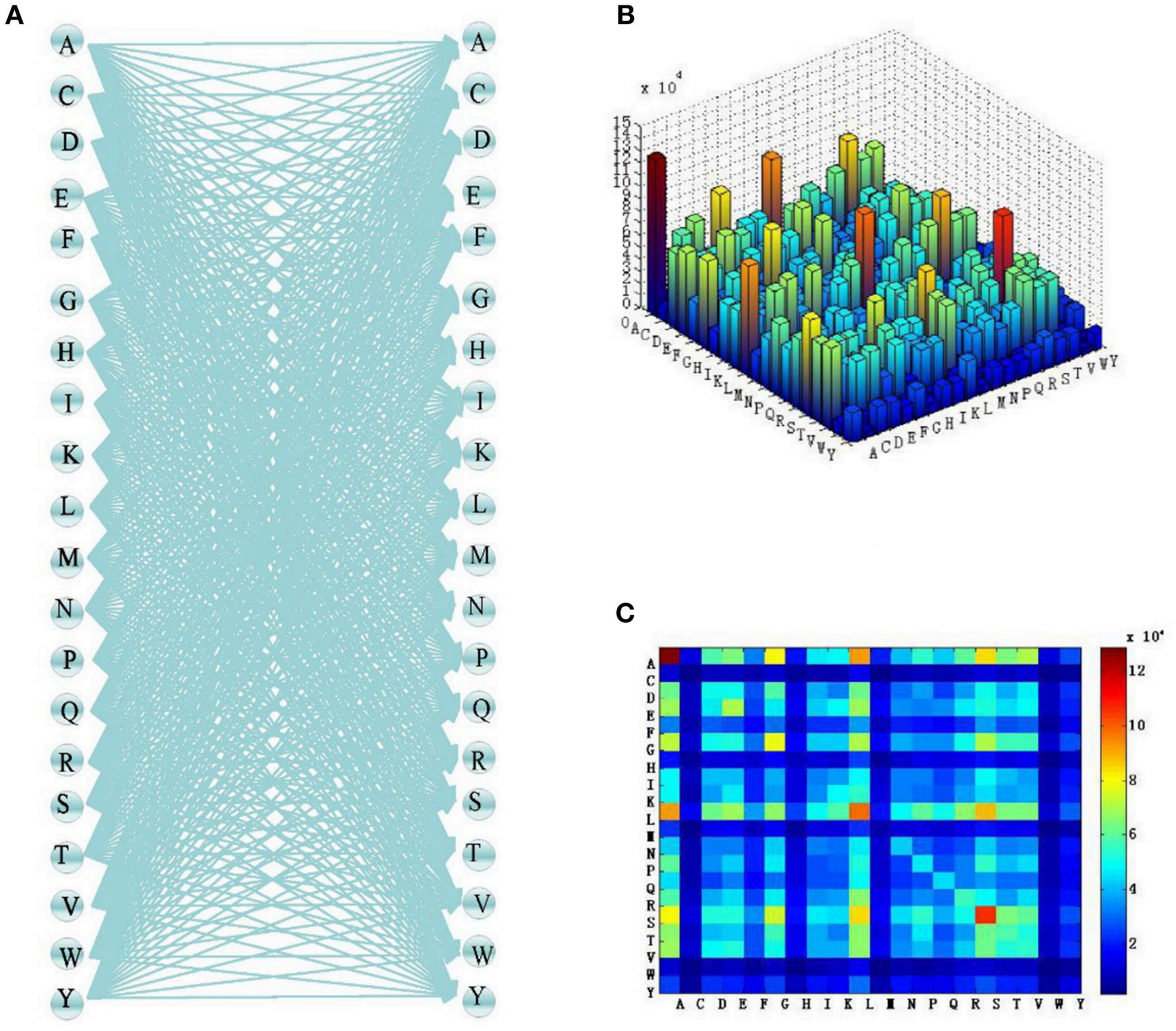
Two-two combination process of amino acids. **(A)** Two-two combination of residues. **(B)** Three-dimensional heat map of amino acid frequency. **(C)** Heat map of amino acid frequency.

For *n* = 2, the combinations of the 20 most common amino acids and the number of occurrences of each combination in the sample datasets are as shown in Figure 3.

This process is similar to full connection in a neural network. Among the 20 common amino acids, anyone can combine with another amino acid (or itself) in pairs, and the combination is random. In the same way as full connection, this leads to overfitting when there are too many data. Therefore, *n* should not be too high when using an adaptive k-skip-n-gram method. When *n* = 1, we have the traditional n-gram model proposed by Guthrie et al. (2006), which does not apply to shorter protein sequences. Therefore, *n* was set to 2 in this study.

In this feature extraction method, the combination set of two specified interval amino acids (Wei et al., 2017a) is given by:

(9) $$\begin{array}{l} \{\begin{array}{c} skip\;(K=0)=\left\{A_{1}A_{2},A_{2}A_{3},\cdots ,A_{L-1}A_{L}\right\}\\ skip\;(K=1)=\left\{A_{1}A_{3},A_{2}A_{4},\cdots ,A_{L-2}A_{L}\right\}\\ \vdots \\ \;skip\;(K=k)=\left\{A_{1}A_{2+k},A_{2}A_{3+k},\cdots ,A_{L-k+1}A_{L}\right\} \end{array} \end{array}$$

In addition, *C* is used to represent a set of two amino acids that are combined at all intervals in a sequence (Wei et al., 2017a). Namely:

(10) $$\begin{array}{l} C_{skipgram}=\left\{\cup_{d=0}^{k}skip(K=d)|d=1,2,3,\cdots k\right\} \end{array}$$

Finally, the feature extraction formula (Wei et al., 2017a) is:

(11) $$\begin{array}{l} FV=\left\{\frac{N(a_{m1}a_{m2}\cdots a_{mn})}{N(C_{skipgram})}|1\leq m_{i}\leq 20,1\leq i\leq n\right\} \end{array}$$

Where *N*(*C*_*skipgram*_) is the total number of elements in set *C*,*a*_*m*1_*a*_*m*2_⋯*a*_*mn*_ are the 20^*n*^ kinds of amino acid combinations of length *n*, *N*(*a*_*m*1_*a*_*m*2_⋯*a*_*mn*_) is the frequency that the two-two combination in *a*_*m*1_*a*_*m*2_⋯*a*_*mn*_ occurs in *C*_*skipgram*_

#### Mixed Representation Algorithm (Seq-Str)

Some researchers have combined different feature extraction methods and achieved very good classification results (Dehzangi et al., 2013; Zou et al., 2014; Leyi et al., 2015, 2018; Chen X. et al., 2016; Ding et al., 2016, 2017a,b; Li et al., 2016; Chen et al., 2017,a,b, 2018c,d,e; Su et al., 2018 Shen et al., 2019; Wei et al., 2019; Zhu et al., 2019). Wei et al. (2015) proposed a novel feature extraction method that uses both the profile of PSI-BLAST (Altschul et al., 1997) and the profile of PSI-PRED (Jones, 1999), which contain rich evolutionary information and secondary structure information, respectively. In this way, the 473-dimensional feature can be extracted.

1. Extract 20-dimensional features based on PSI-BLAST as follows:
  (12) $$\begin{array}{l} \text{FV}=\left\{{\bar{S}}_{i}=\frac{1}{L}\sum_{z=1}^{L}S_{z,i}|\;i=1,2,\ldots 20\right\} \end{array}$$
  *S*_*z, i*_indicates that during the evolution process, the residue at the “z” position in the sequence *S* is mutated to the fraction of the “i” species, and “i” is one of the 20 common residues. *S_i_* indicates that during the evolution, the residue in sequence *S* is mutated to the average score of the ith residue.
2. Extracting 420-dimensional features based on *n*-gram: The Adaptive *k*-skip-*n*-gram algorithm that does not consider the *k* value is the *n*-gram method. Here, take *n* equal to 1 and *n* equal to 2
3. Based on the secondary structure sequence, the following six features are extracted (Wei et al., 2015): Three feature extraction formulas for spatial arrangement
  (13) $$\begin{array}{l} CMV_{H}=\sum_{z=1}^{n_{H}}P_{H_{z}}/L(L-1) \end{array}$$
  Where *P*_*H*_*z*__represents the position index of the zth H in the secondary structure of the sequence *S*. *n*_*H*_ represents the total number of occurrences of H in the secondary structure of sequence.
  Two feature extraction formulas for the percentage of the maximum continuous length (Wei et al., 2015).
  (14) $$\begin{array}{l} Rmax_{C_{H}}=max\;\left\{C_{H}\right\}/L \end{array}$$
  *C*_*H*_ represents the length of the fragment in which H appears consecutively in the sequence of the secondary structure.
  A new feature for distinguishing between two structural classes, α + β and $\frac{\alpha }{\beta }$ : (Wei et al., 2015)
  (15) $$\begin{array}{l} f_{\beta \alpha \beta }=n_{\beta \alpha \beta }/L_{seg}-2 \end{array}$$
  This formula calculates the frequency at which β*αβ* appears in the fragmented sequence *S*_*seg*_, *n*_β*αβ*_ represents the number of times β*αβ* appears in *S*_*seg*_, *L*_*seg*_indicates the length of *S*_*seg*_.
4. Extracting 27 features based on structural probability matrices: Three features from the overall information and 24 features from local information

### Feature Selection

Based on the feature extraction methods described in section Feature extraction, We extracted a 188-dimensional, 400-dimensional feature set based on sequence information, and a 473-dimensional data set based on sequence and secondary structure information representing the entire bacteriophage protein sequence dataset. Some redundant or irrelevant cases were still present in these features. The existence of invalid features wastes time and computational resources, and affects the classification accuracy of the model (Chen et al., 2018b,f,g; Dao et al., 2018; Yang et al., 2018; Zhu et al., 2018a,b). In this paper, the Max-Relevance-Max-Distance (MRMD) (Zou et al., 2016) method was used to select features and identify higher-quality feature sets, i.e., the optimal feature subset. In this method, Pearson's correlation coefficient is used to calculate the correlation between features and class labels (*MR*), thus enabling the selection of features with strong correlation to the target class. Three distance functions (Euclidean and cosine distances and the Tanimoto coefficient) are used to calculate the redundancy between features (*MD*) and identify features with low redundancy.

Taking the two eigenvectors (*X,Y*) as an example, Pearson's correlation coefficient (Pearson, 1909) expressed as follows:

(16) $$\begin{array}{l} \rho_{X,Y}=corr\;(X,Y)=\frac{cov\;(X,Y)}{\sigma_{X}\sigma_{Y}} \end{array}$$

Where σ_*X*_ and σ_*Y*_ denote the standard deviation of the two vectors, *cov*(*X, Y*) is the covariance, which is used to measure the relationship between two random variables. The covariance formula is as follows:

(17) $$\begin{array}{l} cov\;(X,Y)=\frac{\sum_{i=1}^{n}\left(X_{i}-\overset{-}{X}\right)\left(Y_{i}-\overset{-}{Y}\right)}{n-1} \end{array}$$

Where $\overset{-}{X}$ and $\overset{-}{Y}$ denote the mean of the respective vectors.

The formula for the Euclidean distance (Larson and Edwards, 1991; Deza and Deza, 2009) is:

(18) $$\begin{array}{l} ED_{i}=\frac{1}{M-1}\sum_{\;}^{\;}\sqrt{\sum_{q=1}^{n}{\left(x_{q}-y_{q}\right)}^{2}} \end{array}$$

Where *M* is the number of feature vectors,*n* is the total number of elements in each vector, and *x*_*q*_, *y*_*q*_ are the *q*-th elements in *X, Y*, respectively.

The cosine distance formula (Tan et al., 2005) is:

(19) $$\begin{array}{l} COS_{i}=\frac{1}{M-1}\sum_{\;}^{\;}\left(\frac{X\cdot Y}{\left||X||\cdot ||Y|\right|}\right) \end{array}$$

Where

(20) $$\begin{array}{l} \left\|X\right\|=\sqrt{\sum_{q=1}^{n}x_{q}^{2}} \end{array}$$

The Tanimoto coefficient (Rogers and Tanimoto, 1960) is given by:

(21) $$\begin{array}{l} TC_{i}=\frac{1}{M-1}\sum_{\;}^{\;}\left(\frac{X\cdot Y}{||X|{|}^{2}+||Y|{|}^{2}-X\cdot Y}\right) \end{array}$$

Using these distance metrics, we identified the features with the strongest correlation and minimum redundancy with respect to the class labels. In different scenarios, we can increase the weights of *MR* and *MD* (*max*(*wr* × *MR*_*i*_ + *wd* × *MD*_*i*_)) to ensure the acquired features are suitable for the classification task.

## Experiments

### Performance Evaluation Criteria

A 10-fold cross-validation method was employed to evaluate the models. There are four common evaluation indicators, namely the accuracy (*ACC*), sensitivity (*SN*), specificity (*SP*), and Matthews' correlation coefficient (*MCC*) (Feng et al., 2013a, 2018; Chen W. et al., 2016; Wei et al., 2017b,c; Xu et al., 2017; Jingjing et al., 2018). These are expressed as follows (Zou et al., 2013; Chen et al., 2014; Qu et al., 2017):

(22) $$\begin{array}{l} SN=\frac{TP}{TP+FN} \end{array}$$

(23) $$\begin{array}{l} SP=\frac{TN}{TN+FP} \end{array}$$

(24) $$\begin{array}{l} ACC=\frac{TP+TN}{TP+TN+FP+FN} \end{array}$$

(25) $$\begin{array}{l} MCC=\frac{TP\times TN-FP\times FN}{\sqrt{\left(TP+FN\right)\left(TP+FP\right)\left(TN+FP\right)\left(TN+FN\right)}} \end{array}$$

Where TP denotes true positive, i.e., the number of positive samples that are predicted to be positive samples, TN denotes true negative, i.e., the number of negative samples that are predicted to be negative samples, FP denotes false positive, i.e., the number of negative samples that are predicted to be positive samples, and FN denotes false negative, i.e., the number of positive samples that are predicted to be negative samples.

### Classification Effects of Different Classifiers

Experiment 1: This part of the experiment is based on the feature sets of 188, 400, and 473 dimensions extracted by the method in Feature extraction. The accuracy of each classification algorithm before and after using the MRMD feature selection algorithm is presented in Table 1.

**Table 1:** Classification results of three data sets under different classification algorithms.

**Feature_extraction**	**Feature_selection**	**number of D**	**LibSVM (%)**	**Naive Bayes (%)**	**Random forest (%)**
CCPA		188D	68.5	78.3	91.3
	MRMD	185D	68.5	78.3	91.5
AKSNG		400D	60.3	71.8	88.7
	MRMD	252D	60.3	72.8	89.0
Seq-Str		473D	80.6	80.9	92.6
	MRMD	189D	82.0	83.1	93.2

The data in Table 1 indicate that, for the classification of bacteriophage proteins, no matter which feature extraction algorithm is used, whether or not feature selection is performed, the random forest algorithm is the best classification effect.

### Performance of Different Feature Extraction Methods

Experiment 2: Experiment 1 showed that the random forest algorithm produces the best classification of bacteriophage proteins. In this second experiment, the 188-dimensional and 400-dimensional datasets extracted based on sequence information (Seq Based), a 473-dimensional dataset extracted based on structure (Seq and stru Based), and two combined feature sets (Com Based) were integrated into the random forest algorithm, and the resulting performance was compared. The experimental results are presented in Table 2.

**Table 2:** Classification performance under different feature extraction methods.

**Extraction method**	**Number of D**	**SN (%)**	**SP (%)**	**ACC (%)**	**MCC (%)**
Seq based	188D	87.4	93.6	91.3	81.5
	400D	82.8	92.4	88.7	76.1
Seq and str based	473D	86.2	97.2	92.6	85.1
Com based	588D	87.1	93.2	91.2	80.7
	661D	87.5	96.5	93.1	85.3

Feature fusion can boost the recognition performance by combining the complementary information of different features (Zhu et al., 2016, 2018c). A 588-dimensional feature set was obtained by combining the features of the 188- and 400-dimensional feature sets, and a 661-dimensional feature set was obtained by combining the features of the 188- and 473-dimensional feature sets. According to the experimental results, the 188-, 473-, 588-, and 661-dimensional feature set models give better bacteriophage protein classification performance, However, based on the data of the other three evaluation indicators, the 661-dimensional feature set obtained by combining the 188-dimensional feature set extracted based on the sequence information and the features of the 473-dimensional feature set extracted based on the sequence and the secondary structure is the best. This indicates that the feature set extracted by the feature representation algorithm containing both sequence information and structural information in phage protein classification has the best influence on the classification effect, and also shows that combining some feature sets in protein classification is effective for improving classification performance.

### Importance of Feature Selection

Experiment 3: This experiment used the random forest classification algorithm to classify the feature sets after MRMD. The results are given in Table 3.

**Table 3:** Classification performance under each model.

**Model**	**Feature_extraction**	**SN (%)**	**SP (%)**	**ACC (%)**	**MCC (%)**
Mode l	CCPA (188)	87.5	93.4	91.5	81.4
Mode 2	AKSNG (400)	82.9	92.2	89.0	76.0
Mode 3	Seq-Str (473)	86.7	96.6	93.2	84.8
Mode 4	Combine (588)	87.6	93.5	91.5	81.5
Mode 5	Combine (661)	87.9	96.3	93.5	85.3

The comparison of the data in Tables 2, 3 shows that after using the feature selection algorithm (MRMD), the classification effect does not change with the decrease of the dimension, and even with the decrease of the dimension, the classification effect becomes better. After removing the redundant features, the best classification performance is still the data set obtained by feature combination, that is, the 256-dimensional feature set obtained by removing redundant features from the 661-dimensional feature set.

### Comparison With Recent Methods

Experiment 4: To provide an objective demonstration of the performance of the model described in this paper, this experiment compared the optimal proposed model with bacteriophage protein classification models proposed in recent years. The results are presented in Table 4.

**Table 4:** Performance comparison against recent methods.

**Model**	**SN (%)**	**SP (%)**	**ACC (%)**	**MCC (%)**
Feng et al. (2013b)	75.7	80.7	79.1	54.9
Ding et al. (2014)	75.7	89.4	85.0	65.5
Zhang et al. (2015)	87.0	83.0	85.0	70.1
This search	87.9	96.3	93.5	85.3

It is clear from Table 4 that the bacteriophage classification model proposed in this paper achieves a good classification effect, with a classification accuracy of 93.5%. Compared with Feng, it has increased by 14%, compared with Ding and Zhang by 8%. In the other three evaluation indicators, there are also different degrees of improvement, indicating that the model proposed in this paper is an effective tool for phage protein classification.

### Analyzing the Impact of Eight Physicochemical Properties

This section summarizes the first eight dimensional features that have a significant impact on the classification effect of bacteriophage proteins. The top eight features are listed in Table 5 in order of their impact.

**Table 5:** Impact of physicochemical properties on classification.

**NO**.	**Fea name**	**Score**	**Implication**
1	Fea 120	1.0	Position of the 100%th neutral electrical storage amino acid in a sequence
2	Fea 157	0.9968696407744475	Position of the 100%th helical amino acid in a sequence
3	Fea 178	0.9950260206126923	Position of the 100%th soluble amino acid in a sequence
4	Fea 99	0.9949600329187752	Position of the 100%th neutral polarizability amino acid in a sequence
5	Fea 136	0.9948079966447566	Position of the 100%th large tensile amino acid in a sequence
6	Fea 83	0.994509178771573	Position of the 100%th high-electrode amino acid in a sequence
7	Fea 52	0.994137797849692	Position of the 100%th small van der Waals volume amino acid in a sequence
8	Fea 31	0.9937317569946658	Position of the 100%th hydrophilic amino acid in a sequence

According to the information in this table, the effects of eight physicochemical properties of amino acids on the classification of bacteriophage proteins are evenly distributed, and that which has the greatest impact on the classification is the charge property of amino acids.

## Conclusion

Bacteriophage proteins are of special significance for cell typing and pathological research. It is very important to correctly classify virion and non-virion bacteriophage proteins. Therefore, this paper has proposed the following classification model: (1) higher-quality feature datasets are extracted with extraction algorithms based on feature combination; (2) the optimal feature subset is selected using the MRMD algorithm for feature selection; and (3) the random forest algorithm is applied to perform protein classification. The model can achieve accuracy of up to 93.5% for the classification of bacteriophage proteins. This demonstrates that the model developed in this paper is an important tool for the classification of bacteriophage proteins. For the future direction, link prediction paradigms, which have been successfully applied in the prediction of disease genes (Zeng et al., 2017) and miRNAs (Liu et al., 2016; Zeng et al., 2018), can be considered for identification of bacteriophage proteins. It might also be important to integrate evolutionary information using tools like evolutionary trees and networks (Yang et al., 2013, 2014). Finally, computational intelligence such as neural networks (Song et al., 2018a,b) and evolutionary algorithms (Hang et al., 2018) can be applied in this field.

## Author Contributions

XR implemented the experiments and drafted the manuscript. LL and CW initiated the idea, conceived the whole process, and finalized the paper. All authors have read and approved the final manuscript.

### Conflict of Interest Statement

The authors declare that the research was conducted in the absence of any commercial or financial relationships that could be construed as a potential conflict of interest.
